# Supplementary material for: Effect of Eight-Week Strength Training on Body Composition, Muscle Strength and Perceived Stress in Community-Dwelling Older Women
Source: Geriatrics (Basel). 2025 Oct 23;10(6):136. doi: 10.3390/geriatrics10060136 (PMC12641721; doi:10.3390/geriatrics10060136)
Supplement: Supplementary file 1 [file geriatrics-10-00136-s001.zip › geriatrics-3857448-supplementary.pdf]

Supplementary Table 1. Eight-week progressive strength exercise program

Week	Warm-up (10 min)	Main exercises (three sets each)	Reps/Duration	Rest	Progression rules
1–2	A total of 5 min marching in place, arm circles, dynamic stretches	1. Sit-to-stand from chair<br>2. Seated resistance-band row<br>3. Dumbbell biceps curl<br>4. Glute bridge on mat<br>5. Standing lateral leg raises<br>6. Modified plank (on knees)	10–12 reps (or 20–30 s hold for core)	60 s	Start with light band / 0.5–1 kg dumbbells. Borg RPE 11–12 ("light–somewhat hard").
3–4	Same warm-up	1. Partial squat to chair<br>2. Standing band row<br>3. Dumbbell lateral raises<br>4. Hip bridge on stability ball<br>5. Side-lying hip abductions<br>6. Side plank (knees)	12–15 reps (25–35 s hold)	60 s	Progress to stronger band or +0.5 kg weight when >15 reps achievable at RPE <13.
5–6	Same warm-up	1. Bodyweight squat (no chair)<br>2. Band chest press<br>3. Seated dumbbell overhead press<br>4. Stability ball hamstring curl<br>5. Step-ups (low step)<br>6. Plank with alternating leg lift	12–15 reps (30–40 s hold)	60–75 s	Progress by band color, weight increment (0.5–1 kg), or exercise variation. Target RPE 12–13.
7–8	Same warm-up	1. Squat + overhead press ("sit-to-stand + press")<br>2. Band row with external rotation<br>3. Biceps curl + shoulder press<br>4. Bridge march on stability ball<br>5. Forward lunges (assisted if needed)<br>6. Side plank with hip lift	12–15 reps (35–45 s hold)	75 s	Maintain RPE 12–14. Increase band resistance, add dumbbell weight, or introduce combined/unstable variations.
